# Differences in Tobacco Use Among a Sample of At-Risk Chinese, Filipino, and Vietnamese Adult Men Living in the SF Bay Area

**DOI:** 10.1177/1179173X19867947

**Published:** 2019-08-12

**Authors:** Daniel E Toleran, Robynn S Battle, Phillip Gardiner

**Affiliations:** 1 Asian American Recovery Services, Inc., South San Francisco, CA, USA; 2 Prevention Research Center, Oakland, CA, USA; 3 Social and Behavioral Sciences/Neurosciences and Nicotine Dependence, Tobacco Related Disease Research Program, University of California, Oakland, CA, USA

**Keywords:** Chinese, Filipino, Vietnamese, tobacco, other tobacco

## Abstract

**Background:**

Smoking among Asian men has been studied, but differences in tobacco and cigarette use among US- and non-US-born Asian subgroups, especially those at risk for substance use or sexually transmitted diseases, has not been well-studied.

**Aims:**

To learn about the smoking of cigarettes or blunts among Asian ethnic groups, and whether place of birth, age, or primary language spoken at home is associated with smoking.

**Methods:**

Study participants were 125 adult (age > 18 years) Chinese, Filipino, or Vietnamese men living in San Francisco, Daly City, or San Jose, California, who self-reported substance use in the past 30 days. Information collected included sexual orientation, past year contact with the criminal justice system, place of birth, and primary language spoken at home. Bivariate analyses were used to compare the differences in self-reported smoking of cigarettes or tobacco-marijuana blunts by ethnicity, age, place of birth, and primary language spoken at home.

**Results:**

Filipinos had significantly higher rate of cigarette use (51%; *P* = .02) and smoking blunts (28%; *P* = .02) compared with Chinese (23% and 5%, respectively) or Vietnamese (34% and 17%, respectively); US-born Filipinos also had more days of cigarette use in the past 30 days (16 days; *P* = .05) compared with Chinese (8 days) or Vietnamese (6 days) participants.

**Conclusions:**

This study found differences in self-reported rates of cigarette and blunt use among Asian ethnic groups which suggest opportunities for targeted interventions. Future studies of tobacco or blunts use for these largely immigrant groups should take into account country of birth and language spoken at home in developing tobacco prevention services for this population.

## Introduction

There is a dearth of studies about smoking behaviors among Asian American adult men who have sex with men (MSM) or those with a history of serving jail time for drug-related charge. Studies have principally reported on heterosexual Asian American men and suggested differences in smoking rates among various ethnic groups, and have sometimes had conflicting findings. From a study using National Adult Tobacco Survey data, Mukherjea et al^1^ reported high prevalence of smoking among Japanese, Korean, and Filipinos (18.8%, 15.3%, 13.6%, respectively). Earlier studies used data from the California Health Interview Survey. An et al^2^ found a high prevalence of smoking among adult Korean, Vietnamese, and Filipinos (36.7%, 32.4%, 24.5%, respectively) compared with South Asian, Japanese, and Chinese men (15.6%, 15.0%, 14.6%, respectively). Tang et al^3^ observed a high prevalence of smoking among Korean and Vietnamese men (35.9%, 31.6% respectively), compared with the aggregated rate for Asian Americans (13.8%). Lee et al^4^ found Filipino men had higher smoking rates than both their Chinese peers (24% vs 14%) and overall among Asian American men (21%). Lee et al^5^ in their review study involving sexual minority men and their tobacco use found none reported information about the ethnicity of Asian American participants.

There have been 2 reports on the use of marijuana in combination with tobacco among Asian Americans. Both reported on heterosexual young men of Cambodian heritage living in Richmond or Oakland, California.^6,7^ For this group, the study suggested “smoking” includes the use of cigarettes, cigars, and blunts (marijuana in hollowed out cigars) as a social construct that was applied somewhat interchangeably among its members.

Furthermore, many studies cited above analyzed data from state or national datasets which often do not distinguish between those who are United States or foreign born, whether English is the primary language used at home, or their immigrant status. Baluja et al^8^ suggest this information may be valuable because disaggregating health statistics by race/ethnicity, immigrant status, and among immigrant populations their country of birth can enhance the development of targeted smoking prevention programs.

California has the second largest Asian American population proportionally in the United States,^9^ but due to the practice of aggregated data reporting, their diversity is often not reported. From a program developer’s perspective, this lack of information about the identified groups limits their ability to design targeted and responsive intervention or smoking prevention programs. The present study has two aims: first, to assess differences in smoking prevalence (cigarettes, cigars, and blunts) among at-risk Chinese, Filipino, and Vietnamese men identifying as MSM or having contact with the criminal justice system; second, to determine if primary spoken language is associated with smoking in this population.

This brief report discusses findings from Project 3-3-3 (P3), a prevention program developed by Asian American Recovery Services, Inc. (AARS) and designed to reduce substance use, HIV and hepatitis C virus (HCV) infection among Chinese, Filipino, and Vietnamese men, focusing on MSM and other men at risk in San Francisco, San Mateo, and Santa Clara counties in California. P3 was developed in response to high rates of HIV and HCV infection and high participation in substance abuse treatment programs among those belonging in the target population as reported by the Department of Public Health in the respective counties. The program was a 6-module group-level intervention; an earlier publication provides additional information.^10^ Although use of multiple substances (alcohol, tobacco, marijuana, and other drugs) was reported across all P3 participants, this study focused on past 30-day smoking, including blunts.

## Methods

Participant recruitment took place in San Francisco, Daly City, and San Jose, cities which are in San Francisco, San Mateo, and Santa Clara counties, respectively. Using a mix of purposive and snowball sampling, P3 staff members engaged participants through community outreach at ethnic-specific cultural events, local nightlife venues, and peer support groups for individuals involved with the criminal justice system due to drug-related offenses. Social media networking sites such as Facebook and Craigslist were also used to promote the program and recruit potential participants. Baseline, exit, and 3-month follow-up data were collected from 125 individuals; only the baseline data were used for this analysis.

Eligible participants met all the following 4 criteria: (1) Chinese, Filipino, or Vietnamese men; (2) self-reported substance use in the past 30 days; (3) at risk for HIV or HCV; and (4) live or socialize in San Francisco, Daly City, or San Jose, California.

### Data collection

The survey was approved for its procedures through its National Institutes of Health (NIH) recognized Institutional Review Board. The data came from the National Outcome Measures Survey (NOMS), a grant requirement by the Substance Abuse and Mental Health Services Administration’s Center for Substance Abuse Prevention (SAMHSA/CSAP).^11^ National Outcome Measures Survey comprised 135 questions with 6 distinct sections: (1) demographics; (2) cigarette, alcohol, and substance use; (3) family and personal relationships; (4) sexual behaviors; (5) HIV and hepatitis knowledge; and (6) health care and access. Baseline data from section (1) demographics, including age and ethnicity, and section (2) on past 30-day use of cigarette and other tobacco were used in this analysis.

### Measures

The outcomes of interest were self-reported prevalence of smoking among ethnic groups in the past 30 days of cigarettes (yes or no) or other tobacco (yes or no; type). Among prevalent smokers, participants were asked the number of days in the past month (0-31 “days of use”) they smoked cigarettes or other tobacco, and what other tobacco was used (eg, cigars, blunts).

Demographic data including ethnicity, age, length of time in the United States, and the primary language spoken were collected by self-report through a prescribed survey instrument. Only Chinese, Filipino, or Vietnamese participants were included for this analysis as they were the primary population for the program. Participant age was dichotomized as 18 to 29 versus ⩾30 years. Nativity was categorized as United States or foreign born. Primary language spoken was categorized as English or not English.

### Analysis

Chi-square tests were used to compare the differences in self-reported smoking or use of other tobacco by ethnicity. Additional analysis included 1-way analysis of variance (ANOVA) tests between ethnicity and days of use. Binomial logistic regression tests were run to examine possible associations between ethnicity and cigarette or other tobacco product, controlling for age, place of birth, and primary spoken language.

## Results

Participant characteristics: 98% of the participants answered all survey questions. Participants (N = 125) had a mean age of 33.2 years and were of Chinese (n = 39), Filipino (n = 51), or Vietnamese (n = 35) descent (Table 1). The group was predominantly MSM (70%), and a higher percentage of Filipinos (37%) were involved in the criminal justice system compared with other ethnicities. More than half (59%) of all respondents reported being an immigrant, with a higher percentage of Vietnamese and Chinese respondents reported belonging to this category, χ^2^ = 8.96 (2), N = 124, *P* = .01. Vietnamese were more likely to speak their native language (54%) at home than Filipinos (24%).

**Table 1.** Participant characteristics (N = 125).

Demographic	Chinese n = 39	Filipino n = 51	Vietnamese n = 35	Total cohort N = 125
MSM	72	63	77	70
Criminal justice	28	37	23	30
Nativity—Foreign born	47	56*	79	59
Primary language—Asian	48	24*	54	39
Age group—18-29 years old	44	51	49	48
Age group—30 and older	56	49	51	52

Abbreviation: MSM, men who have sex with men.

* *P* = .05 difference observed between Filipino and Vietnamese.

Filipinos (51%) were significantly more likely than Chinese (23%) or Vietnamese (34%) participants to report prevalent smoking of cigarettes, χ^2^ = 2.56 (2), N = 123, *P* = .02, or blunts, χ^2^ = 7.60 (2), N = 121, *P* = .02. Filipinos also reported a greater number of days smoking cigarettes and blunts relative to Chinese or Vietnamese (P = .05) (Table 2).

**Table 2.** Cigarette or blunts use by ethnicity and age.

Comparison groups	Self-reported substance use in past 30 days (at baseline)			
	Reported use (%)		Days of use, mean (SD)	
	Cigarettes	Blunts	Cigarettes	Blunts
Ethnicity				
Chinese	23*	5*	7.95 (18.81)	1.00 (4.98)
Filipino	51*	28*	16.37 (27.16)**	10.86 (26.86)
Vietnamese	34*	17*	5.91 (10.89)	6.20 (18.28)
Age group				
18-29 years old	43*	15*	8.21 (12.40)	2.05 (6.69)
30 years old and older	33*	19*	7.49 (13.12)	3.73 (9.01)

* *P* = .02; **P = .05.

There were no significant associations found between age and smoking cigarettes or blunts, although more 18- to 29-year-olds when compared with the older group reported more days of smoking than those ⩾30 years (42% vs 33%).

In addition, when we analyzed place of birth and smoking or use of other tobacco, Filipinos (51%) and Vietnamese (34%) respondents had a higher rate of cigarette use compared with their Chinese peers (14%), χ^2^ = 6.88 (2), N = 124, *P* = .02. US-born Filipino respondents also had the highest rate of smoking blunts (28%) compared with their Vietnamese and Chinese peers (0% for each), χ^2^ = 8.43 (2), N = 124, *P* = .05. Interestingly, Filipinos who spoke another language at home, although fewer in number than the Vietnamese sample, were more likely to be cigarette smokers compared with their Vietnamese counterparts.

Filipino participants regardless of place of birth reported significantly more days of smoking in the past 30 days than their Chinese and Vietnamese counterparts, *F*(2, 122) = 3.07, *P* = .05. There were no significant differences between ethnic groups in reported days of use for blunts.

## Discussion

Filipino men had a significantly higher self-reported prevalence of smoking cigarettes and blunts as well as having a greater number of days of use for cigarette and blunts. Filipinos speaking a language other than English were more likely to be smokers compared with their Vietnamese counterparts. Findings specific to cigarette smoking are consistent with earlier studies suggesting that adult Filipino men have comparable or higher prevalence of smoking compared with other Asian ethnic groups.^2,4,12^

Based on our findings for place of birth, it suggests the importance of in-language prevention programs for both Filipino and Vietnamese immigrant individuals. In addition, it also suggests identifying the language or dialect is best suited for the group. Further exploration of the differences between US- and foreign-born Asian Americans is indicated, whether the difference is consistent involving a larger sample size, and if other factors in addition to language such as time lived in the United States or age at the time of immigration influence smoking cigarettes or blunts.

Among Asian Americans in the San Francisco Bay Area, the use of blunts among Filipinos is a new finding and adds to our current knowledge about Cambodian men and their use of blunts. As both Filipino and Cambodian men reported use of marijuana combined with tobacco, we recommend further studies to determine if this a persistent trend for these Asian ethnic groups in the region. As the first study to explore blunts use among both immigrant and US-born Asian Americans, we recommend continuing to build the body of knowledge about these communities and their use of cigarettes and blunts to focus on intervention design for these populations.

The present study also differentiates the broad-brush representation of Asian American male tobacco use provided by general population surveys. In focusing on at-risk populations, such as those who are MSM or have had criminal justice involvement, the findings offer nuanced information about the diversity within Asian American communities and their tobacco use.

Limitations to the study include its small sample size and its focus on at-risk subpopulations, and cannot be generalized to all Chinese, Filipino, and Vietnamese MSM or those who have criminal justice involvement in the San Francisco Bay Area. The use of purposive and snowball sampling may have led to the recruitment of participants from similar peer or social groups and may have resulted in having shared characteristics. Finally, the NOMS survey instrument contained a very limited number of measures on smoking-related behaviors such as whether they were past smokers or participated in a smoking cessation programs, and there were no measures relevant to immigrant populations such as language spoken with friends and family members, and English language fluency and comprehension. Additional questions might have offered insights about other environmental factors that may be influencing these respondents’ smoking behaviors. This study supports the need to disaggregate data for Asian American ethnic groups to the subgroup level in support of developing prevention programs tailored to an indicated population at-risk for tobacco-related disease.
